# Treatment and Training of the Deaf Soldier

**Published:** 1918

**Authors:** 


***Treatment and Training of the Deaf Soldier.*** By DUNDAS GRANT, Major, R. A. M. C. Abs. from the *Recalled to Life*, April, 1918.

The author states that his aim with regard to the blind has been to put them in the way of providing against poverty and privation, whereas in dealing with the deaf, he has aimed to restore to them the power of understanding and communicating with their fellows.

Lip reading is by far the most practical means of putting the deaf man into social communication with others and of making him eligible for a large number of occupations.

Such instruction is given in Paris at the Institution Nationale des Sourds-Muets in the rue Saint-Jacques. The system seems to have been started at Lyons, the chief center of the XIV th region. There is also such instruction at the convalescent hospital in Bordeaux. In talking with some of the patients in the institutions, the author noticed that it appeared more difficult for patients who knew both English and German to understand English than it was to understand German. This difficulty, he believes, was due to the restricted lip movement in English. A patient detected the author's foreign accent in French, but he could understand all that was said.

Mr. Haycock states that lip-reading with a free use of the blackboard is the means of communication in his classes in Edinburg, and that in private conversation writing on paper takes the place of the blackboard. The amount of writing necessary varied with individuals. All of the patients objected to using the finger alphabet.

A mental attitude of freshness and cheerfulness is of great importance, and therefore a vivacious and sympathetic manner is a requisite in any teacher. Lip-reading requires very great mental concentration and hence requires that the pupils should come to their lessons fresh, and that they should be provided in the meantime with light and agreeable recreations. The first lessons are the most trying, for until the patient gains some ease in lip-reading, the mental strain is very great. Many of the patients are not at all accustomed to such intense mental activity.

